# Exploring Medical Students’ Perspectives on Artificial Intelligence in Medical Education: A Knowledge, Attitude, and Practice Study Conducted at Jinnah Medical College, Pakistan

**DOI:** 10.7759/cureus.97453

**Published:** 2025-11-21

**Authors:** M Hassaan Shah, Muhammad Faizan, Farooq Akbar, Hooriya Nasir, Abdul A Saddiqui, Yasir Fazal, Lalunu Abid, Syed M Haider Maroof, Samar Hasnain, Sana Bibi, Hoorain Nasir, Fatima Bibi

**Affiliations:** 1 General Medicine, Khyber Teaching Hospital, Khyber Medical College, Peshawar, PAK; 2 Medicine, Jinnah Medical College, Peshawar, PAK; 3 General Surgery, Lady Reading Hospital, Peshawar, PAK

**Keywords:** artificial intelligence, attitude, knowledge, medical students, practice

## Abstract

Objective: The present research aimed to investigate medical students’ views regarding the use of artificial intelligence (AI) in medical education and to study its impact on their annual examination percentages.

Study design and setting: This was a cross-sectional study conducted at Jinnah Medical College, Peshawar, Pakistan.

Participants: A total of 200 medical students from various academic years were enrolled in the study using a convenience sampling technique. Students who declined to provide consent were not included in the study.

Results: Within the study population, 52 (26%) reported having a high level of knowledge regarding AI, whereas 55 (27.5%) considered their knowledge to be limited. While 166 (83.5%) participants acknowledged awareness of AI applications, only 52 (26%) had received formal education or training in AI during their medical studies. Additionally, 142 (71%) participants believed that AI has the potential to enhance medical education, and 118 (59%) reported prior use of AI tools for study or research purposes.

Conclusion: This study highlights that although medical students have a strong awareness of the use of AI, they lack in-depth understanding and formal training. The findings emphasize the need to integrate structured AI education into medical curricula to prepare future healthcare professionals for effective and safe use of AI in academic and clinical practice.

## Introduction

Artificial Intelligence (AI) is the field of computer science focused on enabling machines to carry out tasks that typically require human intelligence. These tasks include processing visual inputs, understanding spoken language, and translating between languages [1].

Systems that exhibit AI differ from those demonstrating natural human intelligence. These AI-driven systems are designed to mimic human-like decision-making, learning capabilities, and problem-solving strategies [1].

AI is increasingly used to automate complex functions such as analyzing data, identifying patterns, and generating user-specific feedback. By reducing the time and effort involved in repetitive or information-heavy tasks, AI contributes to increased efficiency and ease of use. In the education sector, AI has become an influential tool for student support and academic success. With the emergence of advanced conversational tools such as ChatGPT (developed by OpenAI, San Francisco, CA) and Bard (developed by Google, Mountain View, CA), AI's role in learning has grown significantly. It has captured both public and academic interest.

The integration of AI into various fields, including medicine, is expanding. Medical specialities such as dermatology, radiology, ophthalmology, pathology, psychiatry, cardiology, oncology, gastroenterology, and neurology are actively adopting AI-powered tools for improved diagnostics and workflow efficiency [2]. Use of AI is also rising among students; a 2023 global survey revealed that 40% of students have already used AI to support their academic work [3]. Another study from the same year found that 53% of students reported using generative AI platforms as private tutors to assist in learning [3]. In medical education, students are showing growing trust in AI’s potential. A survey of 1,000 dental students and surgeons found that 60% of participants believed their curriculum should be revised to include competencies related to AI [4]. AI-powered assistants simulating patients have been used to improve healthcare students’ counselling and diagnostic skills [5,6].

AI can be particularly beneficial for medical students by simplifying complex terminologies through personalized mnemonics, generating concise summaries of difficult topics, and offering interactive learning tools that reinforce understanding. Machine learning and deep learning are components of AI that contribute to medical education [5]. The developed world has already inculcated AI in its educational system, but the developing countries still face a lot of challenges in its implementation.

Having a working knowledge of AI is essential in the modern era. As the world undergoes a rapid technological transformation, medical education must also adapt by incorporating AI into its curriculum. This integration is vital to prepare the next generation of healthcare professionals to effectively engage with AI-driven tools in clinical decision-making, diagnostics, and research. Understanding how medical students currently perceive and engage with AI is crucial for shaping effective educational strategies.

This study is particularly important in the context of Pakistan, where, despite having a vast and expanding medical education sector, there is a noticeable lack of research and data on the awareness, attitude, and practical engagement of medical students with AI. As global medical systems increasingly adopt AI technologies, it is essential that Pakistani institutions do not lag behind. Therefore, the primary aim of this study was to assess medical students’ knowledge, attitudes, and practices (KAP) regarding AI in medical education. The secondary aim was to identify any significant associations between students’ KAP regarding AI and their academic performance.

## Materials and methods

Study design

This was a cross-sectional study conducted to assess KAP regarding AI among medical students and to determine any association with their academic performance.

Study population and sampling

Medical students of Jinnah Medical College, Peshawar, were eligible for inclusion. Students from other medical colleges and paramedical programs were excluded. The total population of eligible students was 400. A sample size of 200 was calculated using Cochran’s formula for finite populations, assuming a 95% confidence level, 5% margin of error, and an estimated 50% proportion for KAP variables. Participants were recruited using a convenience sampling technique.

Questionnaire development

Data were collected using a structured questionnaire prepared by a team of investigators. The questionnaire was reviewed by faculty members and pretested in a pilot study involving 20 students from the same institution. Based on the pilot study, minor modifications were made to clarify wording and improve comprehensibility. The final questionnaire included demographic information (age, gender, and year of study), academic performance (score in the last professional examination), and questions assessing knowledge, attitudes, and practices regarding AI in medical education. The full questionnaire is provided as Appendix A.

Reliability and validity

The internal consistency of the questionnaire was assessed using Cronbach’s alpha, which yielded a value of 0.82, indicating good reliability. Face and content validity were established through faculty review.

Ethical considerations

The study was approved by the Ethics Board of Jinnah Medical College (reference: JMCP/ERC/2025-004). Informed consent was obtained from all participants. Anonymity was maintained by not collecting personal identifiers such as names, contact details, or email addresses. Participants could withdraw at any stage.

Data collection

The finalized questionnaire was distributed among participants over a period of one month. Completed questionnaires were checked for completeness and internal consistency before data entry.

Data analysis

Data were entered and analyzed using IBM SPSS Statistics version 26 (IBM Corp., Armonk, NY). Descriptive statistics were applied to summarize participants’ demographic characteristics and responses related to knowledge, attitudes, and practices regarding AI in medical education. Results were presented as frequencies and percentages for categorical variables, while means and standard deviations were used for continuous variables where applicable.

The chi-square (χ²) test of independence was performed to determine associations between categorical variables, such as students’ academic grades and their use of AI-based tools, prior formal training, and engagement in AI-related educational activities. Statistical significance was set at p < 0.05.

For quality assurance, data were checked for completeness and internal consistency before analysis. Cross-tabulations were also generated to visualize associations between key variables. Missing data were handled by excluding incomplete responses from specific analyses while retaining the remainder of the dataset.

## Results

Demographics

The study included 200 respondents, comprising 118 (59%) male and 82 (41%) female participants. The highest number of participants, 80 (40%) students, were from the first year of medical school, followed by 58 (29%), 43 (21.5%), and 19 (9.5%) from the third, second, and fourth years, respectively. Participants' ages ranged from 17 to 24 years, with the most common age being 21 years, represented by 55 (27.5%) students.

A total of 22 students (11%) had yearly grades above 80%, while 151 students (75.5%) scored between 60% and 80%. Only 13.5% of students had grades below 60%. The highest reported percentage was 86%, and the lowest was 51% (Table 1).

**Table 1:** Sociodemographic characteristics of the respondents (N = 200)

Characteristic	Frequency (n)	Percentage (%)
Gender		
Male	118	59.0
Female	82	41.0
Year of study		
First year	80	40.0
Second year	43	21.5
Third year	58	29.0
Fourth year	19	9.5
Age range	17–24 years	

Knowledge of AI

Out of 200 respondents, 197 (98.5%) had heard of the term AI. When asked to rate their knowledge of AI, 93 (46.5%) participants described it as moderate, 40 (20%) as high, and 12 (6%) as very high. Meanwhile, 32 (16%) participants rated their knowledge as low, and 23 (11.5%) as very low.

Only 107 (53.5%) participants actually identified AI as the simulation of human abilities, such as learning, reasoning, problem-solving, and perception. In contrast, 83 (41.5%) participants confused AI with Internet search engines, while 10 (5%) believed AI to be an electronic device. Only 46 (23%) participants correctly identified AI in the context of medical education as the use of computer-based tools that help students learn, adapt, and make decisions through intelligent data analysis.

About 166 participants (83%) claimed that they know how to use AI. While responding to a question assessing their knowledge of specific AI tools, about 139 (69.5%) participants reported awareness of ChatGPT. Only four (2%) participants identified IBM Watson. Similarly, only 18 (9%) participants recognized Copilot, whereas a total of 69 (34.5%) participants were aware of Meta AI. Only about six (3%) and 16 (8%) participants identified Amazon Web Services AI and Microsoft Azure, respectively.

When asked about familiarity with AI technologies used in medical education, such as machine learning, deep learning, language processing, and computer vision, 33 (16.5%) participants were very familiar, 129 (64.5%) participants were somewhat familiar, while 38 (19%) participants were not familiar at all.

Only 52 (26%) participants had received formal education or training in AI during their medical studies (Table 2).

**Table 2:** Knowledge and awareness of AI (N = 200) AWS: Amazon Web Services

Item	Frequency (n)	Percentage (%)
Heard of AI		
Yes	197	98.5
No	3	1.5
Self-rated knowledge		
Very low	23	11.5
Low	32	16.0
Moderate	93	46.5
High	40	20.0
Very high	12	6.0
Ability to use AI		
Yes	166	83.0
No	34	17.0
Definition of AI		
Correct (simulation of human abilities)	107	53.5
Search engine	83	41.5
Electronic device	10	5.0
AI in medical education definition		
Correct	46	23.0
Incorrect/Other	154	77.0
Awareness of specific AI tools (N = 200)		
AI tool	Aware (n)	Aware (%)
ChatGPT	139	69.5
IBM Watson	4	2.0
Copilot	18	9.0
Mera AI	69	34.5
AWS AI	6	3.0
Microsoft Azure	16	8.0
Familiarity with AI technologies and formal training (N = 200)		
Item	Frequency (n)	Percentage (%)
Familiarity with AI technologies		
Very familiar	33	16.5
Somewhat familiar	129	64.5
Not familiar	38	19.0
Formal training in AI		
Yes	52	26.0
No	148	74.0

Attitude towards AI in medical education

A total of 142 (71%) participants believed that AI can enhance medical education, while 28 (14%) doubted the idea and 30 (15%) were unsure. When asked about their perception of AI's role in improving teaching methods and learning outcomes, 159 (79.5%) participants responded in a positive manner, while 41 (20.5%) gave a neutral response. Additionally, 156 (78%) participants supported the integration of AI into traditional teaching methods such as lectures and textbooks. A total of 141 (70.5%) believed that AI could enhance students' academic performance, and 170 (85%) agreed that more awareness about AI should be promoted among medical students. Regarding the factual reliability of AI-generated answers, 122 (61%) participants considered them to be factual. Lastly, 165 (82.5%) participants supported the idea that medical colleges should organize seminars and conferences to promote AI awareness (Table 3).

**Table 3:** Attitudes toward AI in medical education (N = 200)

Attitude Item	Category	Frequency (n)	Percentage (%)
Belief AI can enhance medical education	Yes	142	71.0
	No	28	14.0
	Unsure	30	15.0
Improves teaching methods/outcomes	Very positive	64	32.0
	Positive	95	47.5
	Neutral	41	20.5
Support integration with traditional teaching	Yes	156	78.0
	No	44	22.0
Belief AI enhances academic performance	Yes	141	70.5
	No	59	29.5
Promote awareness among students	Yes	170	85.0
	No	30	15.0
AI-generated answers are factual	Yes	122	61.0
	No	28	14.0
	Unsure	50	25.0
Support seminars/conferences on AI	Yes	165	82.5
	No	35	17.5

Practice of AI in medical education

Of the participants, 118 (59%) had used AI tools for studying or research purposes. Sixty (30%) participants reported using AI for educational purposes daily, 59 participants (29.5%) monthly, while 7 (3.5%) had never used it. A total of 136 (68%) participants used AI for learning and studying, 39 (19.5%) for research and development, 31 (15.5%) for preparing for exams, 20 (10%) for clinical activities, and 14 (7%) for creating presentations.

Regarding their experience with AI, 57 (28.5%) participants stated that it had provided them with incorrect or misleading information during their medical education. In contrast, 82 (41%) participants did not report any such incident.

Additionally, 135 (67.5%) participants expressed interest in attending workshops or training programs focused on AI (Table 4).

**Table 4:** Practice of AI in education (N = 200)

Item	Frequency (n)	Percentage (%)
Used AI tools		
Yes	118	59.0
No	82	41.0
Usage frequency		
Daily	60	30.0
Weekly	59	29.5
Monthly	10	5.0
Rarely	64	32.0
Never	7	3.5
Use cases (multiple response)		
Learning/Studying	136	68.0
Research & Development	39	19.5
Exam preparation	31	15.5
Clinical activities	20	10.0
Presentations	14	7.0
Experienced incorrect info		
Yes	57	28.5
No	82	41.0
Unsure	61	30.5
Interest in workshops/training		
Yes	135	67.5
No	65	32.5

The association between medical students’ grades and multiple factors related to the use of AI was assessed using the chi-square test. Significant associations were observed for the use of AI in medical and research purposes, prior formal training in AI, and the use of AI for learning and studying, as shown in Table 5. Among students scoring above 75%, 43.1% reported using AI-based tools for studying or research in medical education compared to 66.4% of those scoring between 60% and 75%. This association was statistically significant, χ²(31, N = 200) = 52.49, p = 0.009, Cramer’s V = 0.51, indicating a moderate effect size. Similarly, 62.7% of students with grades above 75% and 67.2% of those within the 60-75% range reported using AI for learning purposes, with a significant association, χ²(31, N = 200) = 70.22, p < 0.001, Cramer’s V = 0.59, reflecting a strong effect. In contrast, only 35.3% of students with grades above 75% and 21.3% of those within the 60-75% range had received prior training or education in AI, yet this association also reached significance, χ²(31, N = 200) = 48.36, p = 0.024, Cramer’s V = 0.49, suggesting a moderate effect (Table 5).

**Table 5:** Association between medical students' academic performance and AI use in medical education

Variables	Grades (Percentages)			χ² (df, N=200)	p-value	Cramer’s V	Interpretation
	<60%	60%-75%	>75%				
Participants who used AI-based tools or resources for studying or research purposes in medical education	55.55%	66.39%	43.13%	52.49 (31)	0.009	0.51	Moderate effect
Participants who used AI in learning/studying	81.48%	67.20%	62.70%	70.22 (31)	<0.001	0.59	Strong effect
Participants who received formal education or training on AI in medical studies prior to this study.	29.60%	21.30%	35.30%	48.36 (31)	0.024	0.49	Moderate effect

## Discussion

AI refers to the simulation of human intelligence in machines that are designed to think, learn, and solve problems like a human. In healthcare, AI has addressed numerous challenges encountered in the delivery of medical services, including those related to diagnosis, treatment, and patient care. As AI solutions continue to develop, the ongoing use of AI is expected to expand in medical education as well.

Most of our study participants were male first-year medical students, which was in contrast to studies conducted in Jordan and Nepal, where the majority of participants were female, predominantly from the final year, with ages mostly between 21 and 22 years [6,7].

Our study stated that 197 (98.5%) of students were aware of AI, which was similar to a study conducted in Zambia [8]. Regarding self-assessment of AI knowledge, 40 (20%) participants rated their knowledge as high, and 12 (6%) as very high. This contrasts with a study conducted in Syria, where 70% of participants reported having sound knowledge of AI [9]. Similarly, a study conducted in Pakistan found that 71.3% of participants had fundamental knowledge of AI [5]. One hundred and seven (53.5%) medical students who participated in the study correctly defined AI as the simulation of human abilities, while the rest were not able to identify the correct definition of AI, as seen in other studies [10]. The majority of respondents, 166 (83%) participants, believed they could use AI tools effectively. Participants’ knowledge of specific AI tools aligned with a similar study conducted in Pakistan, with ChatGPT being the most well-known and widely used compared to other AI tools in both studies [11].

Only 52 (26%) participants reported receiving formal education or training in AI during their medical education. This low level of formal training among medical students has also been observed in similar studies conducted in Saudi Arabia, India, and Syria [12, 13, 9].

Overall, participants' attitude towards AI in medical education was optimistic, with 142 (71%) participants believing that AI can enhance medical education and 159 (69.5%) expressing confidence that it can improve teaching methods and learning outcomes. Similar positive attitudes were reported in a study from Western Australia, where 89.9% of participants were optimistic about AI's impact on medicine [14]. The majority of participants agreed that there should be greater awareness of AI among medical students. Similarly, a nationwide survey of healthcare students in Saudi Arabia highlighted strong agreement across disciplines on the integration of AI into the curriculum, reflecting a widespread demand for increased awareness and education [15].

A significant proportion of participants, i.e., 122 (61%), believed that AI-generated responses are factual. This aligns with findings from a study conducted in Kerala, India, where 72% of medical students believe that AI enhances the precision of decision-making and reduces the likelihood of error, thereby enhancing confidence in AI's reliability [10].

Furthermore, the majority of participants, 165 (82.5%), supported the idea that medical colleges should organize seminars and conferences to promote AI awareness, reflecting a growing recognition of the importance of AI education in medicine [10].

Regarding utilizing AI tools, most of the participants, i.e., 136 (68%), reported using AI for learning and academic purposes, whereas 39 (19.5%) used it for research and development. A systemic review on healthcare students’ knowledge, attitude, and skills related to AI indicated that most studies reported a limited level of proficiency in AI usage [16]. Moreover, only 31 (15.5%) participants turned to AI for exam preparation, which aligns with the results of a study conducted in Jordan where 20.9% used AI for the same purpose [6].

Less than 10%, i.e., 20 participants, used AI tools in clinical practice, which contrasts significantly with the study conducted in India, where 40% used AI tools in clinical settings [13]. These findings are concerning because despite a positive attitude, medical students are underusing AI for preparing exams and in clinical settings; this can be attributed to a lack of formal education and training.

Around 57 (28.5%) of participants reported that AI provided them with incorrect or misleading information for their medical studies at least once. Recent findings suggest that AI models can yield clinically inaccurate or misleading results. An exploratory case study evaluating ChatGPT's patient instruction reports for three commonly prescribed medications found that, although the core content was generally accurate, critical safety details were often omitted, and inaccuracies with the potential to cause patient harm were identified [17]. Furthermore, Bhattacharyya et al. demonstrated that 93% of literature references produced by ChatGPT in response to medical questions were either fabricated or inaccurate, highlighting the model’s tendency to generate “hallucinations” when citing literature sources [18]. These findings raise serious concerns regarding the reliability of AI-generated content in medical contexts, particularly when used as a primary source of information. The issue is compounded by the fact that many students may lack the skills necessary to critically appraise AI outputs, making them vulnerable to accepting incorrect data at face value.

Our study further explored the relationship between participants’ grades (percentages) and various factors. No association implying causality can be established between AI use and academic performance. Potential confounding factors (e.g., motivation, digital access, prior exposure) may have influenced both AI engagement and academic outcomes. Future longitudinal or experimental studies are warranted to explore these relationships. This is consistent with the findings of Sakelaris et al. [19], who reported that AI programs for medical students neither positively nor negatively impacted grades. One possible explanation is the lack of formal training in AI, as reflected in our results, where only about 23 (35%) students of those with grades above 75% and 15 students (21.3%) of those within the 60-75% range had received prior training or education in AI. This interpretation is supported by the study of Blanco et al., which highlighted that students with formal AI training demonstrated greater confidence and used AI in a more structured and purposeful manner [20].

To address these challenges, it is essential to adopt a cautious and informed approach when integrating AI into medical education. Students should be encouraged not to rely solely on AI tools for learning or clinical decision-making. AI-generated information must always be cross-referenced with standard textbooks, peer-reviewed literature, and established clinical guidelines, as AI is still evolving and prone to errors. Formal training in digital literacy and critical appraisal should be incorporated into medical curricula to equip students with the skills needed to assess the accuracy and relevance of AI outputs.

Additionally, medical universities should consider developing their own AI tools specifically designed to align with their curricula, examination formats, and recommended textbooks. Such institutionally curated AI systems could provide more reliable, structured, and accurate educational support, minimizing the risk of misinformation while enhancing students’ academic and clinical preparation.

Strengths and limitations

The study addresses a novel and timely topic by exploring medical students’ knowledge, attitudes, and practices regarding artificial intelligence in medical education, filling an important gap in the literature, particularly in Pakistan. Data collection and analysis were conducted rigorously, with the reliability of the questionnaire demonstrated by a Cronbach’s alpha of 0.82 and face/content validity established through faculty review and pilot testing. However, the study has several limitations. Convenience sampling from a single medical college may limit the generalizability of the findings to other institutions. The cross-sectional design captures students’ perceptions and knowledge at a single point in time, which restricts the ability to assess changes in KAP toward AI throughout their education or as AI technology evolves. Finally, observed associations between students’ KAP and academic performance do not imply causality, and other confounding factors influencing academic performance were not controlled for.

Recommendations

To enhance the generalizability of future research, studies should recruit larger and more diverse samples, including medical students from multiple colleges and regions. Additionally, future studies employing experimental or longitudinal designs could better clarify the impact of AI knowledge and engagement on academic performance and clinical competencies. Given the growing role of AI in healthcare, medical schools should consider implementing structured curricula that introduce AI fundamentals early in training. Such education should encompass AI’s applications in diagnostics, treatment personalization, and clinical decision support, while also addressing ethical and practical considerations.

## Conclusions

This study highlights that while medical students demonstrate strong awareness of AI, they often lack in-depth understanding and formal education in the field. Although most participants expressed a positive attitude toward AI and its potential benefits in medical training and practice, significant gaps persist in both theoretical knowledge and practical application. Reported instances of misinformation emphasize the critical need for training in the evaluation and responsible use of AI-generated content. These findings underscore the urgent need to incorporate structured AI education into medical curricula, through seminars, formal instruction, and hands-on training, to equip future healthcare professionals with the competencies required to utilize AI effectively and safely in both academic and clinical contexts. While our study shows associations between students’ KAP toward AI and academic performance, further experimental research is needed to confirm causality and guide curriculum development.

## Appendices

Appendix A

Questionnaire

Exploring Medical Students' Perspectives on Artificial Intelligence in Medical Education: A Knowledge, Attitude, and Practice Study at Jinnah Medical College

Note: By proceeding to complete the questionnaire, you voluntarily agree to participate in this research-based study.

BIODATA

Age: _____________________________________

Gender: __________________________________

Year of Medical School: __________________

Previous Professional Percentage: _______

General Instruction: Please answer all questions with sincerity. Choose the options that best describe your knowledge and attitude towards AI. Answer the question and put a check mark (✔) only on one option given below.

Knowledge

1. Have you ever heard the word Artificial Intelligence? a. Negative b. Yes

2. How do you rate your knowledge of Artificial Intelligence? a. Very Low b. Low c. Moderate d. High e. Very High

3. What is Artificial Intelligence? a. Simulation of human intelligence (learning, reasoning, problem-solving, perception) b. An Internet search engine (Google, Bing, etc.) c. An electronic device (Computer, Calculator, etc.)

4. How would you define Artificial Intelligence (AI) in the context of medical education? a. AI involves using computers to help students learn and make decisions in medical school. For example, AI-powered study platforms can personalize learning materials based on individual progress and performance. Additionally, AI algorithms can assist in medical exam preparation by identifying areas of weakness and providing targeted practice questions. b. AI means creating smart tools that analyze medical information and make healthcare better. These tools can interpret complex medical data, assist in diagnosis, and even suggest treatment options, ultimately improving patient care. AI-powered systems can also support medical education by providing virtual patient simulations and interactive learning experiences. c. AI is about making medical school tasks easier by using technology. This includes automating administrative tasks, such as scheduling or record-keeping, and providing virtual simulations for practice. Moreover, AI algorithms can analyze vast amounts of medical literature and research studies to aid students in understanding complex topics and preparing for exams more effectively. d. I don’t know much about AI in medical school.

5. Do you know how to use AI? a. No b. Yes

6. Which of the following AI platforms are you familiar with in the context of medical education? 1. ChatGPT 2. IBM Watson 3. Copilot 4. Meta AI 5. Amazon Web Service AI 6. Microsoft Azure

7. How familiar are you with specific AI technologies used in medical education, such as machine learning, language processing, and computer vision? a. Very familiar b. Somewhat familiar c. Not familiar at all

8. Have you received any formal education or training on AI in medical studies prior to this study? a. Yes b. No

Practice

1. Have you ever used AI-based tools or resources for studying or research purposes in medical education? a. Yes, of course b. No

2. How often do you use AI? a. Daily b. Weekly c. Monthly d. Rarely e. Never

3. For what purpose have you used AI in your medical studies? (Multiple selection) - Learning and studying - Research and analysis - Exam preparation - Clinical support - Making presentations

4. Has AI ever provided you with incorrect or misleading information in your medical studies? a. Yes b. No c. Unsure

5. Would you be interested in participating in workshops or training programs focused on AI in medical education? a. Yes b. No

Attitude

1. Do you believe that AI has the potential to enhance medical education? a. Yes b. No c. Unsure

2. How do you perceive the role of AI in improving teaching methods and learning outcomes in medical education? a. Very positively b. Positively c. Neutral d. Negatively e. Very negatively

3. Do you support the integration of AI into traditional teaching methods, such as lectures and textbooks, in medical education? a. No b. Yes

4. Do you believe AI can help boost medical students’ academic performance or scores? a. Yes b. No c. Unsure

5. Do you believe there should be more awareness regarding AI among medical students? a. Yes b. No c. Unsure

6. Do you think that answers given by AI are factual? a. Yes b. No c. Unsure

7. Do you think medical colleges should organize seminars, conferences, or events to promote more awareness about AI? a. Yes b. No c. Unsure
